# COVID-19 and its effects on endothelium in HIV-positive patients in sub-Saharan Africa: Cardiometabolic risk, thrombosis and vascular function (ENDOCOVID STUDY)

**DOI:** 10.1186/s12879-021-06426-8

**Published:** 2021-07-31

**Authors:** Nandu Goswami, Per Morten Fredriksen, Knut E. A. Lundin, Chidozie Agu, Simiat Olanike Elias, Keolebogile Shirley Motaung, Bianca Brix, Gerhard Cvirn, Harald Sourij, Evelyn Stelzl, Harald H. Kessler, Adam Saloň, Benedicta Nkeh-Chungag

**Affiliations:** 1 Division of Physiology, Otto Loewi Research Center, Medical University of Graz, Neue Stiftingtalstraße 6/D.05, A-8010 Graz, Austria; 2 Divison of Health Sciences, Alma Mater Europea Maribor, Maribor, Slovenia; 3 Department of Biological & Environmental Sciences, Faculty of Natural Sciences, Walter Sisulu University (WSU), Mthatha, South Africa; 4 School of Health Sciences, Kristiania University College, Prinsensgate 7-9, 0152 Oslo, Norway; 5 KG Jebsen Coeliac Disease Research Centre, University of Oslo and Oslo University Hospital- Rikshospitalet, 0372 Oslo, Norway; 6 Management Sciences for Health, Global Fund RSSH Project, Abuja, Nigeria; 7 Department of Physiology, Faculty of Basic Medical Sciences, University College of Medicine, Lagos, Nigeria; 8 Department of Technology Transfer & Innovation, Durban University of Technology, Tromso Annex, Steve Biko Campus, Durban, 4000 South Africa; 9 Physiological Chemistry Section, Otto Loewi Research Center, Medical University of Graz, Graz, Austria; 10 Clinical Division for Endocrinology and Diabetology, Medical University Graz, Graz, Austria; 11 Diagnostic & Research Institute of Hygiene, Microbiology and Environmental Medicine, Medical University of Graz, Graz, Austria

**Keywords:** HIV, Antiretroviral therapy, COVID-19, Cardiovascular risk factors, Vascular endothelial function, Thrombosis

## Abstract

**Background:**

COVID-19 has affected almost every country in the world, especially in terms of health system capacity and economic burden. People from sub-Saharan Africa (SSA) often face interaction between human immunodeficiency virus (HIV) infection and non-communicable diseases such as cardiovascular disease. Role of HIV infection and anti-retroviral treatment (ART) in altered cardiovascular risk is questionable and there is still need to further carry out research in this field. However, thus far it is unclear, what impact the COVID-19 co-infection in people living with HIV (PLHIV), with or without therapy will have. The ENDOCOVID project aims to investigate whether and how HIV-infection in COVID-19 patients modulates the time course of the disease, alters cardiovascular risk, and changes vascular endothelial function and coagulation parameters/ thrombosis risk.

**Methods:**

A total of 1026 patients will be included into this study. Cardiovascular research PLHIV with (*n* = 114 in each of the three recruiting centers) - or without - ART (*n* = 114 in each of the three recruiting centers) with COVID-19 and HIV-negative with COVID-19 (*n* = 114 in each of the three recruiting centers) will be carried out via clinical and biochemical measurements for cardiovascular risk factors and biomarkers of cardiovascular disease (CVD). Vascular and endothelial function will be measured by brachial artery flow-mediated dilatation (FMD), carotid intima-media thickness (IMT) assessments, and retinal blood vessel analyses, along with vascular endothelial biomarkers and cogualation markers. The correlation between HIV-infection in COVID-19 PLHIV with or without ART and its role in enhancement of cardiovascular risk and endothelial dysfunction will be assessed at admission, weekly, at discharge and, 4 weeks post-discharge (if possible).

**Impact of project:**

The ENDOCOVID project aims to evaluate in the long-term the cardiovascular risk and vascular endothelial function in PLHIV thus revealing an important transitional cardiovascular phenotype in COVID-19. The study was registered under clinicaltrials.gov (NCT04709302).

**Supplementary Information:**

The online version contains supplementary material available at 10.1186/s12879-021-06426-8.

## Background

COVID-19 has affected almost every country worldwide with regard to health system capacities and economic burden, so far. The typical clinical presentations of COVID-19 are fever, sore through, cough, dyspnea, abdominal pain and diarrhea (recently reviewed in Pavelic et al. 2021 [1]). Approximately 15% of patients with COVID-19 must be hospitalized and 5% are critically ill, and often develop acute respiratory distress syndrome and need to be admitted to intensive care units [2]. Risk of severe disease increases with age, male sex, and with co-morbidities such as chronic lung disease, cardiovascular diseases (CVDs), and diabetes [3–8]. COVID-19 can also affect the nervous systems and cause loss of smell or taste sensation [1].

Populations in SSA are increasingly facing a double burden of disease involving the interaction between HIV-AIDS and non-communicable diseases such as CVDs [9]. In general, vascular endothelial function can be regarded as a marker of the net harmful effects of cardiovascular risk factors on the vascular wall [10–15]. HIV-infected patients have premature atherosclerosis [16, 17] and increased risk of ischemic heart disease [18]. The cardiovascular risk represented by HIV-infection is moreover affected by anti-retroviral treatment (ART). ART may, among other effects, prompt dyslipidaemic and metabolic changes in patients over to systemic immune activation, stimulation of endothelial inflammation and atherosclerosis [19]. However, a recent meta-analysis showed that evidence for - and against - a role for ART in the development of CVDs remains unconvicing and that more research is necessary [20].

With the current COVID-19 pandemic, which has also spread across Africa, another burden is added. However, thus far it is unclear, what effect the additional COVID-19 co-infection in people with HIV (PLHIV) will have as there are no data yet on how HIV/AIDS, a prevalent diseases SSA, will affect COVID-19 infection rates or outcomes. Currently, there is no proof for a higher COVID-19 infection rate in PLHIV compared to HIV-negatives. However, there is a possibility that HIV infection and/or ART may have an influence on the course of infection. PLHIV who are on ART with a normal CD4 T-cell count and suppressed viral load should not be at an increased risk of severe disease. On the other hand, this theoretically positive situation could be counterbalanced by recent evidence that ART could also have effects on COVID-19 [21]. Furthermore, HIV-infected patients may additionally suffer from co-morbidities, which could pose a greater risk of COVID-19 infection. Currently, we do not have any data about COVID-19 infection in PLHIV in SSA reflecting who in particular is at high risk for infections, complications and fatal outcomes. As COVID-19 associated multisystem inflammation [21], and the way organ damage caused by COVID-19 occurring in patients with COVID-19 is still incompletely understood, current treatment options are limited. There is therefore an urgent need to better understand the risk of severe and fatal COVID-19 outcomes [22], especially in PLHIV (+/− ART).

A severe course of COVID-19 can cause the development of COVID-19–associated coagulopathy, with features of both disseminated intravascular coagulation and thrombotic microangiopathy, resulting in widespread microvascular thrombosis that may involve consumption of coagulation factors [23] and the liver [24]. There seems to be a causal relationship with the inflammatory and reparative processes involving diffuse alveolar damage (DAD), because thrombi are frequently detected in small pulmonary arteries, most presumed secondary to endothelial damage [25]. The damage of endothelium could be due to the direct viral infection of the endothelial cells, which express ACE-2 receptors, or to a host response [26]. Besides, the alveolar fibrin deposition in DAD may affect the local homeostasis between fibrinolysis and coagulation [27]. Alveolar and endothelial damage of smaller vessels may be followed by microvascular pulmonary thrombosis, which could then extend to larger vessels. Additionally, elevated D-dimer has been observed in patients with COVID-19 [24, 28]. It is known that inceased D-dimer is associated with acute pulmonary emboli (APE), deep venous thrombosis (DVT), cancer, peripheral vascular disease, inflammatory diseases and pregnancy. It has been found that patients with COVID-19, including those not on respirators but bedconfined, develop DVT and APE [29] much earlier than expected [28]. Even with usage of prophylactic anticoagulation, autopsy has shown that deaths in COVID-19 may be caused by the thrombosis in segmental and subsegmental pulmonary arterial vessels [24]. The ENDOCOVID project aims to evaluate the degree to which COVID-19 induced inflammation contributes to endothelial function and coagulation changes and cardiometabolic risk in PLHIV (+/−ART). Endothelial function will be evaluated non-invasively via FMD [11] and blood asymmetric dimethyl arginine (ADMA) [30].

### Problem statement

HIV infection is a burden for the cardiovascular system but coinfection of HIV and SARS-CoV-2 in SSA population could be a very big load and could lead to higher menace of cardiovascular health. However, the potential of risk in this population, including early vascular and endothelial changes, is yet unclear. Given the ongoing global pandemic, epidemiological studies to address this knowledge gap is necessary. The aim of this study is to evaluate cardiovascular risk in HIV and COVID-19 populations, which would also allow assessment of current - and future - cardiovascular risk, via vascular endothelial function measurements. The ENDOCOVID project has been designed to give an evaluation of cardiovascular risk in PLHIV and COVID-19 living in SSA. ENDOCOVID builds up on the EndoAfrica study, which is currently being carried out in SSA.

### Aim and objectives

**Fig. 1:**
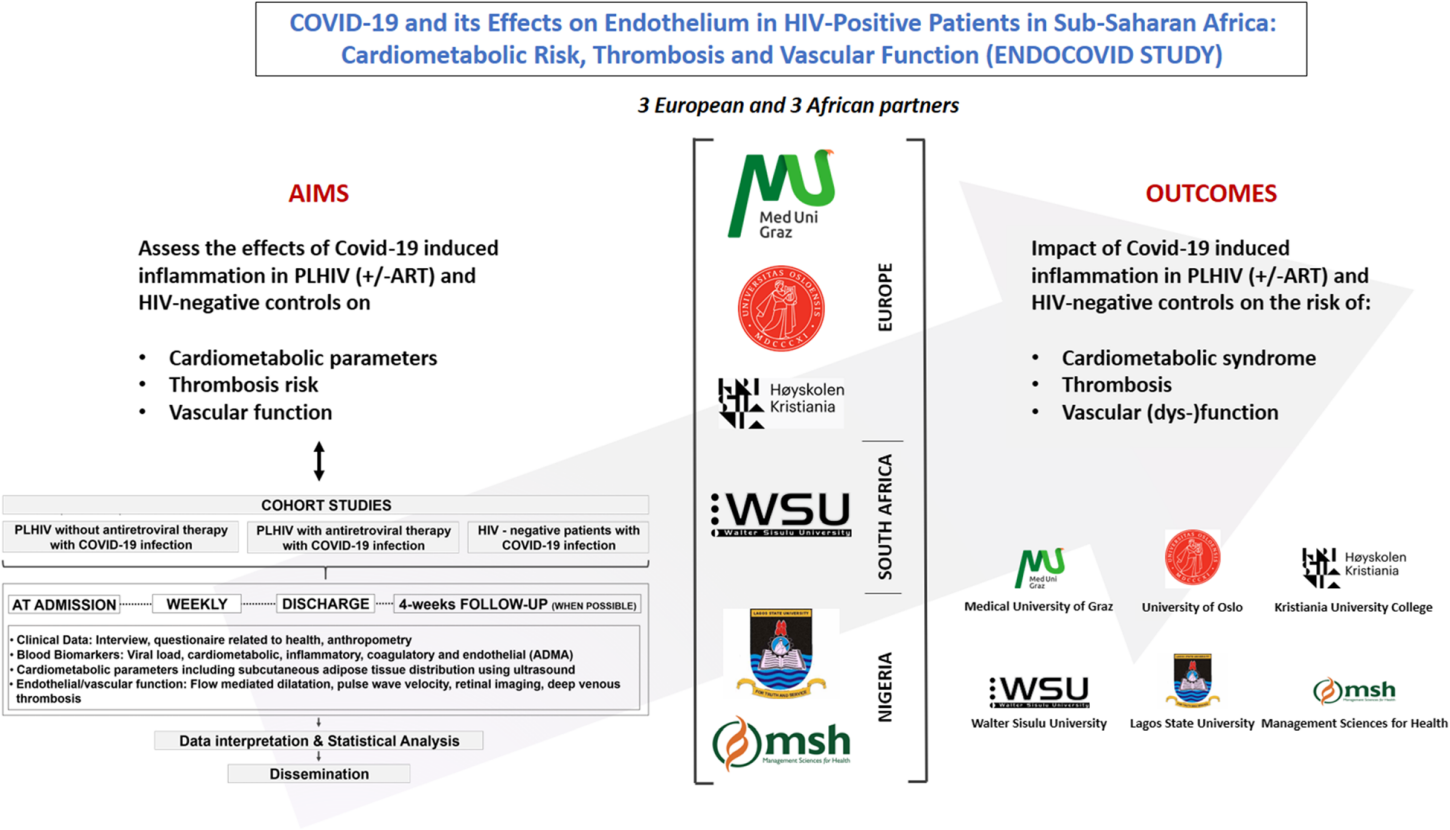
Project aims and objective established for the ENDOCOVID project. Shown are also the partner institutions and the impacts of the project

The general aim of the ENDOCOVID project is to identify whether and how HIV-infection and/or ART in COVID-19 patients relates to the time course of the disease, alters cardiovascular risk, and changes vascular endothelial structure and function in adults living in the SSA (Fig. 1).

Three groups of participants will take part in this project:

1. PLHIV without ART but with COVID-19 (*n* = 114 in each recruiting center)
2. PLHIV with ART and COVID-19 (*n* = 114 in each recruiting center)
3. HIV-negative patients with COVID-19, sex−/age-matched (*n* = 114 in each recruiting center)

The following objectives will be investigated (Fig. 1):

1. Prevelance of PLHIV among those admitted for COVID-19 to clinics in South Africa and Nigeria.
2. Assessment of the incidence of acute respiratory distress syndrome, admission to ICU, fatal course of disease (30-days mortality), and thromboembolic events in hospitalized patients included in the study.
3. Evaluation of the influence of ART on severity of COVID-19 in PLHIV (with or without ART) as well as clinical presentation, changes of endothelial/vascular function and biomarkers.
4. Evaluation of the effects of COVID-19 on endothelial/vascular function and thrombotic factors over infection and after recovery.
5. Correlation of hospitalization and mortality of COVID-19 infected PLHIV (with or without ART) and HIV negatives with co-morbidity incidence;
6. Assessment of the incidence of obesity and insulin resistance in the 3 groups.

## Methods/design

### Study design

**Fig. 2:**
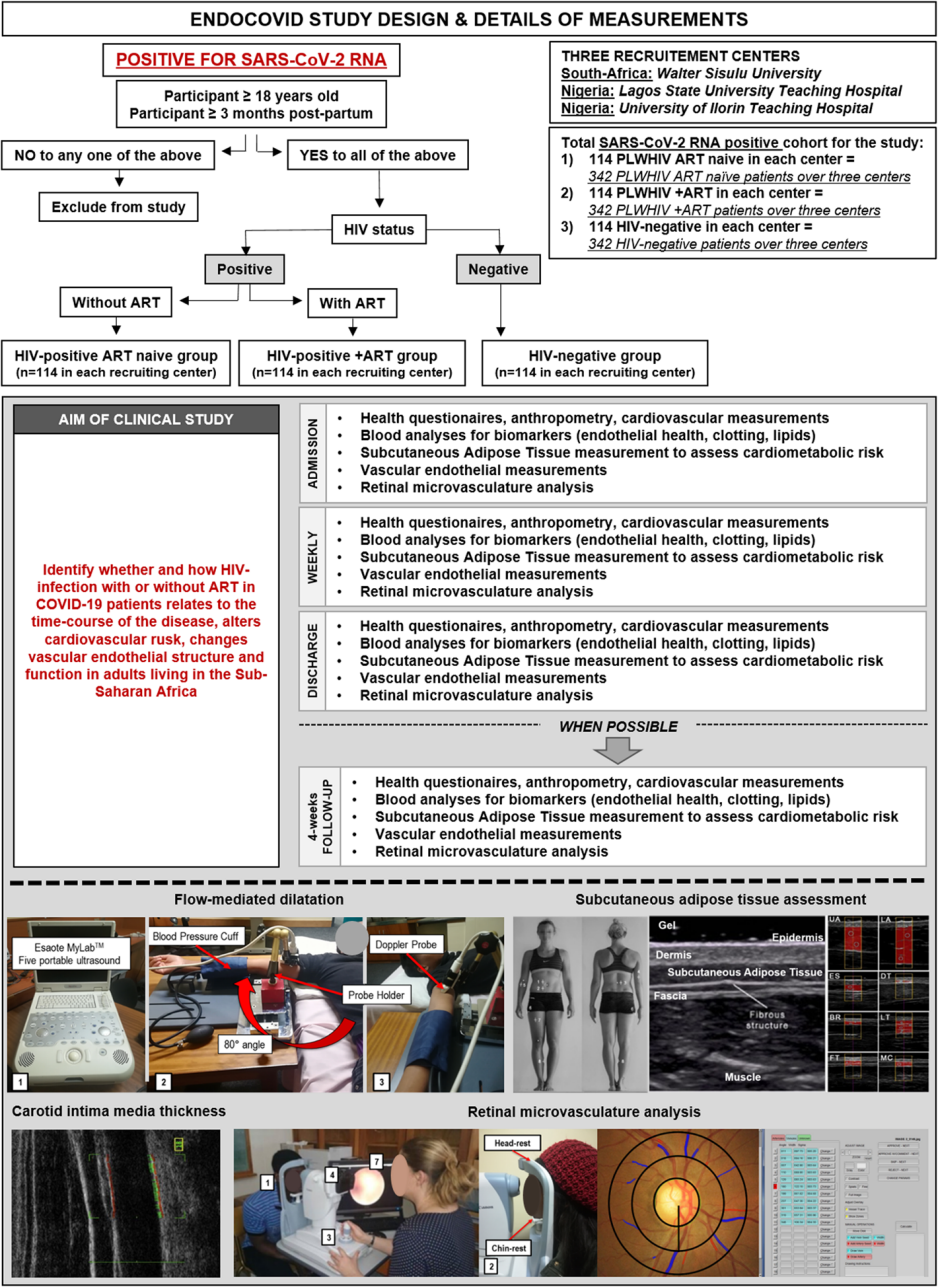
Study protocol and the proposed measurements. While every effort will be made to collect post-discharge data, unplanned and unpredictable lockdowns could prevent post-discharge data collection (hence the use of the term “when possible” during post-discharge follow-up measurements)

In the prospective ENDOCOVID study, monitoring will be carried out at admission (baseline), weekly, at discharge and, a follow-up 4 weeks post-discharge (if possible) (Figs. 1 and 2). The four- week post-discharge collection may be hampered by unpredictable lockdowns; therefore, this measurement may not be carried out in all the participants (that is why it is indicated as “when possible”). Measurements will include health questionnaires, anthropometric measurements, a cardiovascular physical examination, blood collection, Ultrasound-based vascular and endothelial measurements, and fundus imaging for retinal blood vessel analysis (Fig. 2). Results obtained at admission (baseline), weekly, at discharge and, 4 weeks post-discharge (if possible) (Fig. 1) will be compared. ENDOCOVID (protocol version 1 from August 13, 2020) has received ethics approval from Walter Sisulu University, South Africa (EC_202011_006 and 053/2020), as well as University of Illorin Teaching Hospital, Nigeria (NHREC/02/05/2010) and Lagos State University Teaching Hospital, Nigeria (NHREC04/04/2008). Protocol modifiers will be submitted to the respective ethics committees as well as to the clinical trial registration. Verbal and written informed consent will be obtained by the study clinician from each participant prior to being included into the study.

The study was registered under clinicaltrials.gov: NCT04709302, 13 January 2021, *https://clinicaltrials.gov/show/NCT04709302*.

### Participant recruitment and study groups

#### Inclusion criteria

Participantsof the ENDOCOVID study population are patients with COVID-19, who at the same time are infected with HIV (with or without ART), or are HIV negative. Some may be admitted in the ICU. Participants will be recruited by qualified and trained researchers at hospitals based on pre-determined inclusion criteria. For inclusion into the study, participants must be positive for SARS-CoV-2 RNA and 18 years or older. Participants will be excluded from the study if they are less than 3 months post-partum and those with other co-infections than HIV such as tuberculosis (Fig. 2). Participants fulfilling the criteria will be invited into the study and asked to provide written informed consent.

Presence of SARS-CoV-2 RNA will be determined by real-time PCR. Subsequently, potential participants with unknown HIV status will be tested for HIV-infection via a rapid HIV test. A negative HIV test will be used to assign the participants to the HIV-negative group and a positive HIV test to the HIV-positive group, which is further split into two sub-groups according to the ART status of participants. Firstly, participants without ART as the “HIV-positive ART naïve” group. However, it has to be taken into consideration that, the majority of participants in this sub-group will start with ART, soon after recruitment. The third group are patients diagnosed as “HIV-positive on ART”. Therefore, we can evaluate cardiovascular risk and early endothelial changes in both the untreated and treated groups. SARS-CoV-2 RNA, CD4+ T-cell count, and HIV RNA viral load of the HIV-positive participants will be determined at admission (baseline), weekly, at discharge and, 4 weeks post-discharge (if possible) (Figs. 1 and 2). Participants are also required to refrain from smoking, drinking coffee or alcohol, or doing exercise for 4 h prior to the study. The menstrual phase of each female will be recorded.

### Assessments: clinical and laboratory investigations

#### Health questionnaire, cardiovascular measurements, blood chemistry

General information will be obtained from a health questionnaire which include details about cardiovascular health including family and personal history of CVD, medication, smoking, and alcohol consumption (for detailed information, please find the full questionnaire in the supplementary material). Additionally, personal data about participants and their demographic characteristics based on the National Cholesterol Education Program Adult Treatment Panel III (NCEP ATP III) will be collected. Aspects related to the usage of medication, presence of co-infections and HIV disease characteristics (first diagnosis and duration of HIV infection, past and current regimens of ART treatment, viral load, nadir/current CD4+ T-cell count) will also be recorded [31]. Cardiovascular risk factors will be screened through analyses of (i) Lipid profile, (ii) glucose, (iii) glycated hemoglobin (HbA1c), (v) liver function enzymes, and (vi) high sensitivity hs-CRP, (vii) heart rate, (viii) blood pressure and (ix) body anthropometrics [32]. Together with evaluation of SARS-CoV-2 and HIV infection, these measurments in the participants will allow identification of common cardiovascular risk factors: smoking, alcohol consumption, overweight/obesity, hypertension, diabetes mellitus, dyslipidemia, renal impairment, inflammation, and liver disease.

#### Anthropometric assessment

Anthropometric measurements include body mass (BM), body height (h), sitting height (s), and the circumferences of waist and hip. Measurement of height will be performed by a Seca 217 portable stadiometer (Seca, Hamburg, Germany) and measurement of BM (in underwear) will be performed using a Seca 862 electronic scale (Seca, Hamburg, Germany). Body mass index (BMI) is computed as kg/m^2^ [33]. Waist circumference will be measured at the mid-point between the lower costal border, 10th rib, and the iliac crest, using a Lufkin W606P flexible steel tape (Apex Tool Group, Sparks Glencoe, MD, USA). Anthropometric measurements will be carried out according to the International Society for the Advancement of Kinanthropometry [34].

#### Subcutaneous Apidose tissue measurement to assess cardio-metabolic risk

Ultrasound is the most exact technique for thickness measurements of subcutaneous adipose tissue (SAT) layers. This method was recently standardised using eight sites to display SAT and distinguish between fat and present fibrous structures (the trunk (three), the arms (two), and the legs (three)). For calculating mean SAT thickness, it is necessary to carry out repeated series measurements of randomly selected sites of these eight standardized sites [35–37]. Advantages of using this method are that it avoids compression artifacts, distinguishes between fat tissue and neighbouring structures, is not invasive, does not use ionizing radiation, and is easily applicable in the field. The usage of this recent method in overweight and obese groups has recently been reported [36]. Ultrasound imaging is based on the pulse-echo technique whereby a series of ultrasound pulses (each several wavelengths long) is directed into the tissues. While ultrasound systems for diagnostics purposes utilize 1540 ms-1 for distance determination in soft tissues, in adipose tissues 1450 ms-1 [35] is used for measurement of thickness. To minimize fat compression errors, the ultrasound probe is normally placed on the measuring site without any pressure and on a thick layer (5 mm) of ultrasound gel between the probe and the skin. Ultrasound measurements will be performed according to the standardized ultrasound measurement approach [35].

#### Flow mediated dilation (FMD)

FMD is non-invasive technique, ideal for evaluating vascular endothelial functions. Measurement of vascular endothelial functions by FMD has been validated from a prognostic point of view, as well in successful monitoring of disease progression/regression in clinical studies [38–41]. We have also used it previously to assess the cardiovascular risk in PLHIV/AIDS in SSA in the ongoing *EndoAfrica* Study [39].

The FMD procedure is performed as described in detail in the *EndoAfrica* study [39]. Briefly, images of the right radial artery are obtained in B-mode with an Esaote MyLabTMFive US and 12 MHz linear probe (Esaote, Italy). Doppler mode is used to visualise and locate the radial artery with pulse repetition frequency (PRF) set at 6.7 kHz. In pulse wave mode, the angle of insonation is set to + 60°. Radial artery diameter and shear rate measures are recorded continuously with FMD Studio and Cardiovascular Suite version 2.8.1 software (Quipu, Italy), which makes use of automatic edge detection technology. The measurements begin with 1 min baseline recording (rest phase). The blood pressure cuff is then inflated to 50 mmHg above the systolic blood pressure value and it remains at this level for 5 min (ischaemic phase). After 5 min, the cuff is deflated leading to hyperaemia and increased shear rate inducing endothelium-dependent dilatation of the radial artery (post-ischemic recovery phase). The software calculates baseline, and maximum artery diameter, recovery artery diameter, FMD % (difference between maximum, and baseline diameter expressed as %), baseline shear rate and maximum shear rate.

#### Carotid intima-media thickness (IMT)

Carotid IMT is an indirect sonographic assessment of the degree of atheromatous vascular diseases. IMT is a biomarker of arterial wall thickening and therefore also atherosclerosis [42–45]. Enhancements in IMT associate with prevalent and incident cardiovascular morbidity and mortality, including coronary heart disease [45]. The thickness of the media and the intima of the vessels’ change following many conditions and it can be easily and reliably assessed with ultrasound using the B-mode of the common carotid arteries.

IMT measurements will be performed according to pre-established protocols [46]. Briefly, the participants are laid in supine position and the head tilted 30° to the left to evaluate of the right carotid artery, and 30° to the right for evaluation of the left carotid artery. After visualisation of the carotid artery bifurcation in B-mode, IMT measurements will be carried out on the posterior carotid wall on the right and on the left side, 1 cm from the carotid bifurcation. The measured segment must be free of atherosclerotic plaques and the lumen-intima and media-adventitia interfaces must be clearly defined. At least five sagittal measurements on each side are needed for obtaining the average IMT value. Carotid artery IMT average values greater than 1 mm are considered abnormal. The evaluation of the carotid IMT is carried out using Quality Intima-Media Thickness (QIMT) software. Th QIMT software enables automatic measurements of the intima-media thickness and calculates and records the median ± standard deviation IMT values during several cardiac cycles [45].

#### Pulse wave velocity (PWV)

PWV, a marker of aortic stiffness [47], is most commonly measured as the time it takes a pulse wave to travel from the carotid to the femoral arteries divided by the distance multiplied by 0.8. PWV can be measured by several devices. The non-invasive Vicorder device has been shown to have good reproducibility [48] - even when the assessor has limited experience in its usage [49] - and the results obtained reflect those obtained via invasive central blood pressure measurements [50] and those of SphygmoCor device [48]. Based on the criteria of the ARTERY Society Recommendations [51], the accuracy of PWV recorded by the Vicorder device has been described as “excellent” [52]. Vicorder measurements are performed by trained researchers. Patients lie in a supine position on a bed with the head raised to approximately 30°. PWV is measured by a cuff on the right carotid and the right thigh. The distance between the carotid and femoral arteries is recorded by measuring the length between the suprasternal notch and the mid-point of the thigh cuff. Measurements continue until pressure waveforms over the carotid and thigh are clear and reproducible. Subsequently, all tracings are reviewed, and, if necessary, repeated. Selected are only data that show clear pressure waveform upstrokes.

#### Retinal vessel analyses

Retinal imaging analysis is a procedure which enables us to visualize the microcirculation in the eye consisting of blood vessels that are less than ~ 150 μm and include the smallest resistance vessels. They form an important part of our circulatory system and assessment of these retinal microvasculature can provide an indication of cardiovascular health [40, 53]. Several studies have reported that there is a connection between changes in retinal microvasculature and coronary heart disease [54]. The ratio between the diameter of the retinal arteries and veins (A/V) reflects the predisposition and/or association with hypertension and atherosclerosis [55]. Quantitative features from the retinal images are therefore useful for identifying microvascular changes, which can be used as a predictive tool for assessment of the risk of developing cardiovascular diseases [56, 57].

In our study, a non-mydriatic, hand-held, portable digital retinal camera (Optomed Aurora, Optomed Oy, Oulu, Finland) will be used for collecting retinal images. Dimensions of vessels and microvascular state will be analyzed offline with the semi-automated MONA REVA software (VITO, Belgium). Analysis of retinal images will be performed by a trained person in this field. In order to prevent bias, the participant characteristics will not be provided to the person analyzing the images. The vessel widths and pattern features of both the right and left eye will be performed. The width is calculated from the largest six arterioles and venules which cross a zone of 0.5–1.0 disc diameter from the optic disc margin. Final calculations related to the average diameter of arterioles and venules in the retina are presented as central retinal arteriolar equivalent (CRAE) and central retinal venular equivalent (CRVE), respectively [58]. However, for analysis of retinal microvasculature pattern features such as tortuosity, fractal analysis, and lacunarity, it is necessary to measure all blood vessels crossing a zone of 2x disc diameter from the optic disc margin. A summary of the many features of the retina that can be characterized and calculated is provided by Prabhakara and colleagues [59].

#### Sample collection for SARS-Cov-2 RNA detection

Nasopharynegal swab will be used to obtain nasopharyngeal sample for the qualitative detection of SARS-Cov-2 RNA using real time PCR. Plain urine container will be used to obtain urine from participants for the detection of urine albumin and creatinine. Two collection tubes will be used for blood obtained from the ante-cubital vein: One 9-mL plain tube and another 6-mL EDTA tube. Some blood will then be dispensed into EDTA container for determination of CD4+ T cells and full blood count and viral load, while approx. 7 mls will then be dispensed into plain containers allowed to clot at room temperature, spun at 2000 g for 10 min; the serum will then be separated and cryopreserved at − 80 degree Celsius for analysis.

#### Serum biomarker analyses

Blood samples will be collected at admission (baseline), weekly, at discharge and, 4 weeks post-discharge (if possible), which is described in detail elsewhere [60] (Fig. 1). A number of plasmatic pro-inflammatory markers that play a role in endothelial dysfunction will be measured to supplement the endothelial function assessments done on the participants. Levels of serum cytokine and inflammatory markers, such as VCAM- 1, ICAM-1, TNF-α, TNF-β, and NF-κB will be measured at the same time for each participant using Luminex technology (Luminex Bio-Plex 200 system) [61, 62]. Markers of endothelial function such as ADMA will be measured using commercially available ELISA kits. Measurement of effects of COVID-19 on coagulation will be carried out via coagulation tests, calibrated automated thrombogram, thromboelastometry, impedance aggregometry, and markers of thrombin formation. These measurements are important as an increased level of D-dimer over 2500 μg/L is seen, for example, in all cases of pulmonary embolism [63]. Hence, patients with high levels of D-dimer will undergo CT angiography to visualize any accompanying thrombosis of segmental pulmonary arteries [63]. Screening for cardiovascular risk factors will be performed by measurements of (i) Lipid profile, (ii) glucose, (iii) HbA1c, (v) liver function enzymes, and (vi) high sensitivity, hs-CRP.

#### Data collection, management and statistical analysis

Data from the questionnaires, clinical evaluations, and biochemical analyses will be gathered and managed by Research Electronic Data Capture (REDCap) tools hosted at the Walter Sisulu University [64] (Fig. 1).

Statistical analyses will be performed in collaboration with the Biostatistics Unit in the Centre for Evidence-based Health Care, Walter Sisulu University. Descriptive statistical models (ANOVA, Chi Square) will be used for inter-group comparisons, and associations between independent and dependent variables will be tested by multivariate regression analysis models, taking into account confounding variables such as age, gender, ethnicity, smoking, alcohol consumption, medication, and BMI. For long term changes in the same individual, a repeated measures model approach will be engaged.

Strijdom et al., in their discussion of the *EndoAfrica* study design, suggested that sample sizes of up to 300 participants are sufficient to secure statistical power [39]. Fischer’s formula was also used for calculation of sample size: (n) = Z2P (1-P)D2. Since there are no data on the number of people with coinfection of HIV and SARS-CoV-2, the sample size was calculated using the prevalence rate of PLHIV of 4.9% in SSA (UNAIDS report, 2019) [65]. To achieve statistical significance, 94 HIV-infected persons are required. Since we have 3 groups, we will require a total of (94 × 3) = 282 participants. Due to 20–30% drop out rate, 20 more persons in each group will be added to this number, so that the final number of participants required in this project has similar participant numbers that were recruited in the *EndoAfrica* study (282 + 60 = 342 at each recruiting center). Demographic and background data (e.g., smoking and drinking status, BMI) will also be collected and included in the linear regression models as putative confounders [66].

## Discussion

The described project ENDOCOVID, an extension of the *EndoAfrica* study, will provide new data from the SSA context. No previous longitudinal studies in SSA have been carried out to comprehensively evaluate cardiovascular risk and vascular endothelial changes in COVID-19 patients with and without HIV/AIDS. The study will also provide more information related to the outcomes (severity of cases, ICU admissions, mortality rates) in persons with co-infections. The results will be innovative, as currently little is known about the effect of COVID-19 on PLHIV (+/−ART), especially in SSA. Repeated measurements over time will give us valuable knowledge about the progression or regression of markers of cardiovascular risk and disease in COVID-19 and HIV-infected persons. The information obtained from this project is important for developing guidelines and recommendations towards the management of cardiovascular health in patients with COVID-19 and HIV-infection for key stakeholders, including regional health care authorities. The findings of the ENDOCOVID project will furthermore contribute to the current global knowledge based on COVID-19 and HIV and its association with cardiovascular diseases.

The vascular and endothelial endpoints of the ENDOCOVID project will generate novel research data in the South African context and have big potential to improve the prediction of future cardiovascular events not only during COVID-19 but also for other diseases. While some studies have examined the reported association of endothelial dysfunction in COVID-19 patients, only very little information about SSA populations which are at the same time HIV-infected, is currently available. The use of three independent, non-invasive imaging techniques and the measurement of serum biomarkers related to endothelial health and coagulation improve the novelty of the project and also underline its strengths.

## Dissemination

Topics suggested for presentation or publication will be circulated to the principal investigators of the ENDOCOVID study. As part of community engagement, radio and television programmes as well as the social media (LinkedIn, ResearchGate) will be used. Results will be presented at the EACS: 18th European AIDS Conference (2021), IAS: 11th International AIDS Society Conference on HIV Science (2021), CROI: Conference on Retroviruses and Opportunistic Infections (CROI) (2021) and in journals such as AIDS, Clinical Microbiology and Infection, Lancet HIV. Output from this study will be communicated to the South Arican and Nigerian governments through Ministeries of Health.

### Possible limitations

Although the ENDOCOVID project will provide us a lot of new information, it is associated with several risks for which have been proposed impact alleviation strategies. These risks include:

1. All HIV-infected patients in South Africa must immediately begin with ART and it may limit our ability to recruit ART naïve participants. All of these untreated HIV-infected participants will serve as their own untreated controls during the cycle of our project, and we will be able to observe endothelial changes after initiation of ART.
2. Lack of patients because of current lockdown and low prevalence of COVID-19 in SSA region. This may require that we have to expand our cohorts to more regions. That is why we have included two partners from SSA: South Africa and Nigeria. It has also been seen in most parts of the world that the period following the lockdown is accompanied by an increase in the number of COVID-19 related infections. So, between the two countries, we should have no problem in recruiting the requied number of participants to reach statistical significance for this study.
3. The severity of the disease can limit our collection of samples and data about the patient, especially in those cases where the patients with COVID-19 are in the intensive care units and difficult to access for research purposes. However, we will work with clinicians to ensure that we can also evaluate the patients during acute care. Furthermore, patients at home and discharged from the hospital will also be recruited and their data collected. The complete data collection will be done by motivated healthcare partners of this project.
4. It is possible that at the end of the project, we could be confronted with missing data. As missing data can limit the statistical comparisons across groups, we will reduce this via close collaborations with hospital personnel. In addition, we will take the required measures in the statistical analysis plan to deal with missing data.
5. A possible limitation of this project is that it may not always be possible to collect post discharge data at 4 weeks. This may arise due to issues with patitence compliance and/or lockdowns. Every patient will be advised to report back 4 weeks post-discharge for a follow up assessment. Unpredictable and unplanned lock-downs, however, could make post-discharge data collection difficult.
6. Another obvious limitation related to FMD and IMT measurements is the fact that these measurements are operator dependent and how inter-operator variability. It is also important that same vascular regions are measured in each participant at the 4–6 weeks’ follow-up visit. These risks will be reduced by limiting the number of operators in the research team, and training them intensively, and ensuring strict quality control steps by independent experts in this field.
7. As the ART regimens differs across the recruitment sites, and different types of ART may have different effects, it could potentially influence the data that will be collected across the two SSA countries. When analyzing and interpreting the data from the different sites, details of the ART regimes will also be taken into account.

## Conclusions

ENDOCOVID is an innovative project within the *EndoAfrica* study that examines vascular endothelial change and dysfunction as well as cardiovascular risk during COVID-19 infection in PLHIV. Sub-Saharan Africa has the largest number of patients with HIV infection but only little is known about the prevalence of cardiovascular risk factors and early vascular disease in HIV-infected populations in these regions. More importantly, how co-infection with SARS-CoV-2 in PLHIV impacts cardiovascular health is not known. The ENDOCOVID project aims to fill in the knowledge gaps in this field.

### Supplementary Information

**Additional file 1.**

## Data Availability

Not applicable.

## Abbreviations

AIDS: Acquired immunodeficiency syndrome
ART: Antiretroviral therapy
BM: Body mass
BMI: Body mass index
COVID-19: Coronavirus disease 2019
CVD: Cardiovascular disease
FMD: Flow mediated dilation
HbA1c: Glycated hemoglobin
HIV: Human immunodeficiency virus
IMT: Intima-media thickness
PLHIV: People living with HIV
PWV: Pulse wave velocity
REDCap: Research Electronic Data Capture
SARS-CoV-2: Severe acute respiratory syndrome coronavirus 2
SAT: Subcutaneous adipose tissue
SSA: Sub-Saharan Africa
