# Correction: COVID-19 and its effects on endothelium in HIV-positive patients in sub-saharan Africa: cardiometabolic risk, thrombosis and vascular function (ENDOCOVID STUDY)

**DOI:** 10.1186/s12879-023-08933-2

**Published:** 2024-02-15

**Authors:** Nandu Goswami, Per Morten Fredriksen, Knut E. A. Lundin, Chidozie Agu, Simiat Olanike Elias, Keolebogile Shirley Motaung, Bianca Brix, Gerhard Cvirn, Harald Sourij, Evelyn Stelzl, Harald H. Kessler, Adam Saloň, Benedicta Nkeh-Chungag

**Affiliations:** 1 Division of Physiology, Otto Loewi Research Center, Medical University of Graz, Neue Stiftingtalstraße 6/D.05, A-8010 Graz, Austria; 2 Divison of Health Sciences, Alma Mater Europea Maribor, Maribor, Slovenia; 3 Department of Biological & Environmental Sciences, Faculty of Natural Sciences, Walter Sisulu University (WSU), Mthatha, South Africa; 4 School of Health Sciences, Kristiania University College, Prinsensgate 7-9, 0152 Oslo, Norway; 5 KG Jebsen Coeliac Disease Research Centre, University of Oslo and Oslo University Hospital, 0372 Rikshospitalet, Oslo Norway; 6 Management Sciences for Health, Global Fund RSSH Project, Abuja, Nigeria; 7 Department of Physiology, Faculty of Basic Medical Sciences, University College of Medicine, Lagos, Nigeria; 8 Department of Technology Transfer & Innovation, Durban University of Technology, Tromso Annex, Steve Biko Campus, 4000 Durban, South Africa; 9 Division of Physiology, Otto Loewi Research Center, Medical University of Graz, Neue Stiftingtalstraße 6/D.05, A-8010 Graz, Austria; 10 Physiological Chemistry Section, Otto Loewi Research Center, Medical University of Graz, Graz, Austria; 11 Clinical Division for Endocrinology and Diabetology, Medical University Graz, Graz, Austria; 12 Diagnostic & Research Institute of Hygiene, Microbiology and Environmental Medicine, Medical University of Graz, Graz, Austria

**BMC Infectious Diseases (2021) 21:719**

10.1186/s12879-021-06426-8

The original publication of this article contained an incorrect author name. The incorrect and correct information is listed in this correction article. The original article has been updated.

Incorrect

Adam Salon

Correct

Adam Saloň

