# Supplementary material for: COVID-19 and its effects on endothelium in HIV-positive patients in sub-Saharan Africa: Cardiometabolic risk, thrombosis and vascular function (ENDOCOVID STUDY)
Source: BMC Infect Dis. 2021 Jul 31;21:719. doi: 10.1186/s12879-021-06426-8 (PMC8325201; doi:10.1186/s12879-021-06426-8)
Supplement: Supplementary file 1 — Additional file 1. [file 12879_2021_6426_MOESM1_ESM.pdf]

Register

Study ID ________________________________

Demographics

DEMOGRAPHICS

Firstname ________________________________

Surname ________________________________

House/Flat Number ________________________________

Dwelling type ◯ House ◯ Flat or Complex ◯ Informal housing

Building/Complex Name ________________________________

Street name ________________________________

Suburb ________________________________

For how many years have you lived at current address? ________________________________
((months as decimal e.g. 6 months = 0.6))

Telephone number ________________________________

Medical Background

MEDICAL BACKGROUND

Past

Have you had a heart attack in the past? ○ Yes ○ No

Have you been diagnosed with cancer in the past? ○ Yes ○ No

Have you previously had TB? ○ Yes ○ No

If yes, when did you previously have TB? ____________________
(If patient cannot recall date, enter year (yyyy))

Have you had a stroke in the past? ○ Yes ○ No

Present

Do you have high blood pressure (hypertension)? ○ Yes ○ No ○ Unknown

If yes, what year were you first diagnosed? ____________________

Do you have a heart disease? ○ Yes ○ No ○ Unknown

If yes, what year were you diagnosed? ____________________

Do you have high cholesterol? ○ Yes ○ No ○ Unknown

If yes, what year were you diagnosed? ____________________

Do you have any other long lasting health problems? (For example: kidney stones, arthritis, asthma, bilharzia, malaria) ○ Yes ○ No

If yes, please specify what the health problem is and what year you were diagnosed? ____________________

Do you currently have pulmonary Tuberculosis (TB)? ○ Yes ○ No

If yes, are you on treatment? ○ Yes ○ No

When did you start treatment? ____________________
(If the patient cannot recall date, enter year (yyyy))

Diabetes

Do you have Diabetes ○ Yes ○ No

What type of Diabetes do you have?
- ○ Type I Diabetes (also known as Juvenile Onset or Insulin Dependent Diabetes)
- ○ Type II Diabetes (also known as Non-insulin Dependent Diabetes)
- ○ Don't know

How long ago were you told you have diabetes?

- ◯ < 12 months
- ◯ 1-5 years
- ◯ 6-15 years
- ◯ 15+ years

Which of the following do you use to manage your diabetes?

- ☐ Diet
- ☐ Pills
- ☐ Injection
- ☐ Nothing
- ☐ Other

((tick all that apply))

If other, please specify. ______________________________

Family History

Stroke And Heart Disease

	Don't know	No	Yes, under the age of 60	Yes, over the age of 60	Yes, but I don't know the age
Has your mother had any heart disease?	◯	◯	◯	◯	◯
Has your father had any heart disease?	◯	◯	◯	◯	◯
Has your mother ever had a stroke?	◯	◯	◯	◯	◯
Has your father ever had a stroke?	◯	◯	◯	◯	◯

Diabetes

	Don't know	No	Yes, Type I	Yes, Type II	Yes, but I don't know type
Has your mother had any Diabetes?	◯	◯	◯	◯	◯
Has your father had any Diabetes?	◯	◯	◯	◯	◯

High Blood Pressure and Cholesterol

	No	Yes	Don't know
Has your mother had high blood pressure?	◯	◯	◯
Has your father had high blood pressure?	◯	◯	◯
Has your mother had high cholesterol?	◯	◯	◯
Has your father had high cholesterol?	◯	◯	◯

HIV

Are you HIV positive? ◯ Yes ◯ No

If positive, are you on ART? ◯ Yes ◯ No

Which line of ART are you on? ◯ 1st ◯ 2nd

What is the name of the ART? ______________________

For how long have you been on ART? ______________________
((weeks))

Date you were diagnosed with HIV? ______________________

If date unknown, how long ago approximately were you diagnosed with HIV
◯ In last year
◯ 2 - 5 years ago
◯ >5 years ago
◯ >15 years ago

Family Planning

Do you currently have a baby younger than 3 months? ◯ Yes ◯ No

Are you currently pregnant? ◯ Yes ◯ No ◯ Not applicable

Are you currently breast feeding? ◯ Yes ◯ No

Are you on family planning? ◯ Yes ◯ No

Medications

Do you take any medications? ◯ Yes ◯ No

Which medications do you take?
☐ Beta blockers
☐ Statins
☐ Aspirin
☐ Calcium channel blockers
☐ ACE inhibitors
☐ Other
☐ Anti-inflammatory

If other, specify ______________________

What dosage of aspirin do you take? ______________________
(mg)

General Data

Date subject signed consent	__________________ ((dd-mm-yyyy))
Date and time start of interview	__________________
Date of birth	__________________
Age when signed consent	__________________
Gender	◯ Male ◯ Female
Ethnicity	◯ Black<br>◯ White<br>◯ Coloured<br>◯ Indian<br>◯ Other (please specify)<br>◯ Refused
Ethnicity other	__________________
Please indicate the option that describes your current marital status.	◯ Never married<br>◯ Married<br>◯ Living together<br>◯ Widowed<br>◯ Seperated or Divorced
Field site where patient was recruited	◯ Elsies River<br>◯ Ravensmead<br>◯ Bishop Lavis<br>◯ Durbanville<br>◯ Uitsig<br>◯ Adriaanse<br>◯ Potchefstroom<br>◯ Mthatha<br>◯ Fisantekraal
Field worker taking interview	◯ Charmaine Abrahams<br>◯ Shirley Mc Anda<br>◯ Susan van Zyl

Anthropometry

ANTHROPOMETRY

Total body mass ________________ (kg)

Height ________________ (cm)

Body Mass Index (BMI) ________________

Hip circumference ________________ (cm)

Waist circumference ________________ (cm)

Wasit to hip ratio ________________

BLOOD PRESSURE

Systolic ________________

Diastolic ________________

Arm
◯ Left
◯ Right

Heart Rate ________________ (bpm)

Systolic (2nd reading) ________________

Diastolic (2nd reading) ________________

Arm (2nd reading)
◯ Left
◯ Right

Heart rate (2nd reading) ________________ (bpm)

Systolic (3rd reading) ________________

Diastolic (3rd reading) ________________

Arm (3rd reading)
◯ Left
◯ Right

Heart Rate (3rd reading) ________________ (bpm)

Mean Blood Pressure ________________

Mean Heart Rate ________________

URINE ANALYSIS (Dipstick Parameters)

Glucose

- ◯ Normal
- ◯ 1+ 30
- ◯ 2+ 100
- ◯ 3+ 300
- ◯ 4+ 1000

Protein

- ◯ Negative
- ◯ 1+ 30
- ◯ 2+ 100
- ◯ 3+ 500

Leucocytes

- ◯ Negative
- ◯ 1+ ~10-25
- ◯ 2+ ~75
- ◯ 3+ ~500

Microalbuminuria ______________________

Lifestyle

Cigarette Smoking

Are you a smoker — ◯ Yes currently ◯ In the past ◯ Never smoked

What type of cigarette do/did you smoke? — ◯ Snuf ◯ Tobacco ◯ Dagga

On average, how many cigarettes do you smoke on the days that you smoke? — ◯ More than 20 daily ◯ Less than 20 daily

If you have stopped, how long has it been since you last smoked (months)? — ______________________

Alcohol

Have you consumed an alcoholic drink within the past 12 months? — ◯ Yes ◯ No

How often do you typically drink ? — ◯ Daily ◯ 8 or more days a month ◯ Less than 8 days a month

At what age did you start drinking regularly (at least once a week)? — ______________________ ((answer in years))

What do you drink?

	Yes	No
Beer	◯	◯
Spirits (brandy, vodka, cane etc.)	◯	◯
Red Wine	◯	◯
White Wine	◯	◯
Other	◯	◯

When you drink beer, how many standard units do you typically have on a single occassion? (see reference card). — ______________________

When you drink spirits, how many standard units do you typically have on a single occassion? (see reference card). — ______________________

When you drink red wine, how many standard units do you typically have on a single occassion? (see reference card). — ______________________

When you drink white wine, how many standard units do you typically have on a single occassion? (see reference card). — ______________________

When you drink other, how many standard units do you typically have on a single occassion? (see reference card) and describe. — ______________________

Standard unit reference card

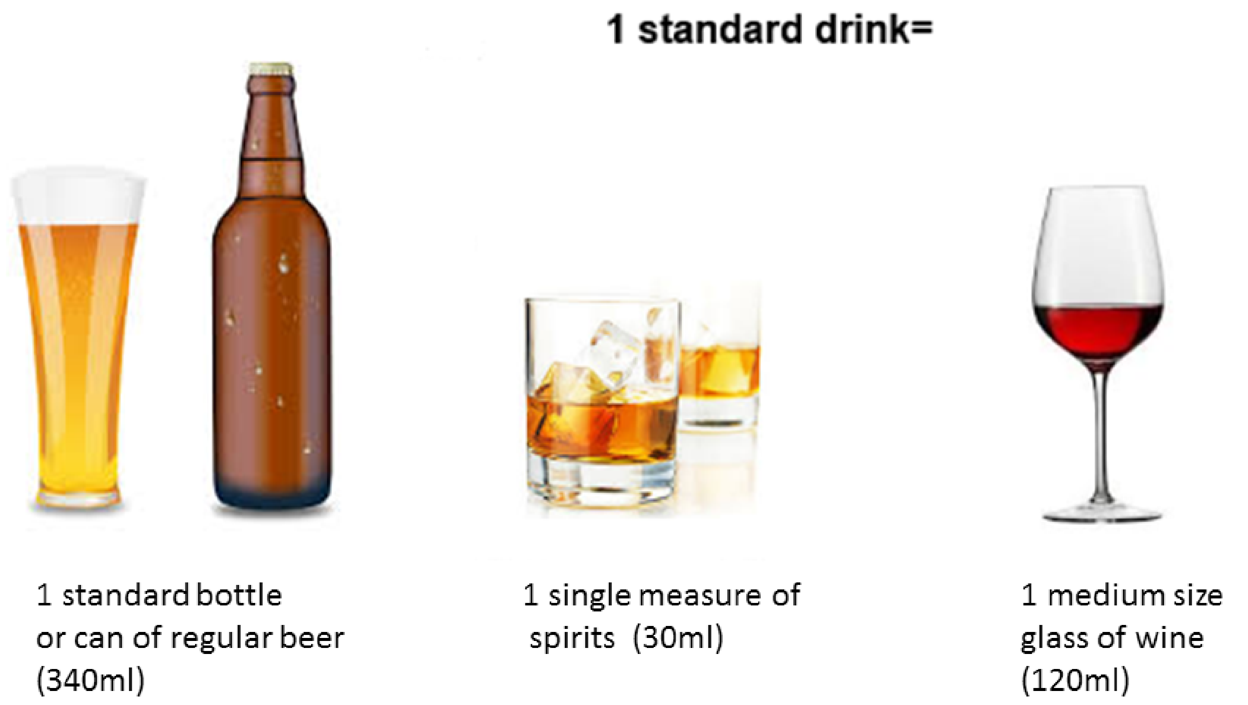

How many drinks to you have on a weekend?

◯ Less than 5 ◯ 5 - 10
◯ More than 10
(Friday night to Sunday night)

Eating Habits

How many meals do you have on a typical day?

◯ One
◯ Two
◯ Three
◯ More than three

Do these meals include fruit and/or vegatables?

◯ Yes ◯ No

Exercise

Are you physically active?

◯ Yes ◯ No

If yes, how many times a week?

◯ Once
◯ Twice
◯ Three times
◯ More than three times

How would you describe the type of exercise you do?

◯ Mild
◯ Moderate
◯ Intensive

Environment

In what type of environment do you live?
- ◯ City
- ◯ Suburb
- ◯ Countryside
- ◯ Industrial
- ◯ Other

If other, please specify ______________________

Do you live on a main road? ◯ Yes ◯ No

Where you live, do you
- ◯ Rent a room in a house
- ◯ Rent a house/ self contained flat
- ◯ Own house/ flat
- ◯ Live with family
- ◯ Not have a usual place to live
- ◯ Live in a shelter (homeless)
- ◯ Live separately (backyard/garden) on a property
- ◯ Informal settlement (shack)

What type of fuel do you mostly use?
- ◯ Electricity
- ◯ Wood
- ◯ Parafin
- ◯ Coal

What is your main source of water for drinking and cooking?
- ◯ Private connection to pipeline
- ◯ Private well
- ◯ Public taps/standpipe
- ◯ Public well
- ◯ Neighbours
- ◯ Water vendor
- ◯ Spring
- ◯ River, stream, lake, pond
- ◯ Rainwater
- ◯ Bottled water
- ◯ Other

Sleeping Habits

On average how many hours of sleep do you get in a 24hr period?
- ◯ 1-3 hours
- ◯ 3-6 hours
- ◯ 6-9 hours
- ◯ > 9 hours

Do you currently do any shift work affecting your sleeping hours? ◯ Yes ◯ No

How well do you sleep?

Bad ____________________ Very well

(Place a mark on the scale above)

Rooibos Tea

Do you drink Rooibos tea? ◯ Yes ◯ No

How many cups do you drink on average day?
- ◯ 1 cup
- ◯ 2-3 cups
- ◯ 3-4 cups
- ◯ >4 cups

How long have you drunk Rooibos regularly?

- ◯ Never
- ◯ Less than a year
- ◯ More than a year
- ◯ My whole life

How do you drink your Rooibos tea?

- ☐ Milk
- ☐ Honey
- ☐ Sugar
- ☐ Lemon

Education

What level of education have you completed?

- ☐ None
- ☐ Primary school
- ☐ High school
- ☐ ABET (Adult Basic Education Training)
- ☐ College/University/Other tertiary institution

(Tick all that apply)

Employment

Which of the following applies to your current employment situation?

- ◯ Unemployed
- ◯ Employed (full time)
- ◯ Employed (part time)
- ◯ Self-employed

As someone who is unemployed, which of the following applies to you?

- ◯ Looking for work
- ◯ Discourage job seeker - not looking for work
- ◯ Student
- ◯ Homemaker
- ◯ Illness/disability prevent me to work
- ◯ Too old to work
- ◯ Other

If other, specify? ________________________________

Income

Do you or someone in your household receive a Government Social Grant?

◯ Yes ◯ No

What is the total of your household income per month?

- ◯ less than R1,000
- ◯ R1,000 - R4,999
- ◯ R5,000 - R9,999
- ◯ R10,000 - R20,000
- ◯ more than R20,000

Lab Sample Form

Sample Date	________________________
Sample Time	________________________ (24 hr annotation)
Time received (lab)	________________________ ((to be completed by lab staff))
Patient Date of Birth	________________________
Gender	◯ Male ◯ Female

SAMPLES (also to be completed by field worker)

Has the patient been fasting for the visit?	◯ Yes ◯ No ◯ Uncertain
Blood {Luminex} = [1x Serum (red cap) 3ml]	◯ Done ◯ Not Done ◯ Under Volume
Blood {Glucose} = [1x Fluoride (grey cap 5ml]	◯ Done ◯ Not Done ◯ Under Volume
Blood {Lipogram & Creatinine} = [1x SST (yellow cap) 7ml]	◯ Done ◯ Not Done ◯ Under Volume
Blood {Hemoglobin & HbA1C} = [1x EDTA (purple cap) 5ml]	◯ Done ◯ Not Done ◯ Under Volume
Blood {Viral load} = [1 x EDTA (purple cap) 5ml]	◯ Done ◯ Not Done ◯ Under Volume
Blood {buffy coat} = [1 x EDTA (purple cap) 10ml]	◯ Done ◯ Not Done ◯ Under Volume
Blood {Coagulation factors} = [1 x Citrate (blue cap) 4.5ml]	◯ Done ◯ Not Done ◯ Under Volume
Blood tubes inverted 8 times	◯ Yes ◯ No
Urine Collected {storage & microA} (approximately 40ml)	◯ Yes ◯ No
Urine Dipstick data completed	◯ Yes ◯ No

HIV STATUS

HIV result	◯ Positive ◯ Negative ◯ Unknown
CD4 Count	________________________ ((from file))

Lipid Profile

LIPID PROFILE

Lipid sample taken
- ◯ Taken
- ◯ Not done (Supply issue)
- ◯ Not done (Too busy)
- ◯ Not done (Refused)
- ◯ Not done (Other reason

Date lipid sample taken ______________________________

Total cholesterol ______________________________

HDL ______________________________

LDL ______________________________

Triglycerides ______________________________

If flags reported, comment ______________________________

Blood Profile

BLOOD PROFILE

Blood for profile taken
- ◯ Taken
- ◯ Not done (Supply issue)
- ◯ Not done (Too busy)
- ◯ Not done (Refused)
- ◯ Not done (Other reason

Fasting glucose ______________________

HbA1c result ______________________
(% (1 decimal))

If HbA1c flag reported, comment ______________________

CRP ______________________

GGT ______________________

FULL BLOOD COUNT

Haemoglobin ______________________
(g/dl (1 decimal))

CD4 count (from patient folder at clinic) ______________________

CD4 count (EndoAfrica result) ______________________

Viral Load ______________________

If FBC flags, comment ______________________

Creatinine

Creatinine sample taken

◯ Taken
◯ Not done (Supply issue)
◯ Not done (Too busy)
◯ Not done (Refused)
◯ Not done (Other reason

Creatinine level ______________________________
(µmol/L (1 decimal))

MDRD eGFR result ______________________________

If flags reported, comment ______________________________

Exclusion

This patient is pregnant. Do no include.

This patient has a baby of 3 months of age or younger. Do not include.

This patient is under 18 year of age. Do not include.

This patient's Hb is too low. Consider for exclusion.

This patient is on 2nd line ART. Consider for exclusion.

Exclude this subject? ◯ Yes ◯ No

Comments ______________________

Flow Mediated Dilatation

FMD files

Operator? __________________________________

Diameter baseline __________________________________
((mm))

Diameter max __________________________________
((mm))

Diameter recovery __________________________________

FMD Percentage __________________________________
((%))

SR baseline __________________________________

SR max __________________________________

Comments __________________________________

SR Area (u.a.) __________________________________

SR AreatoMax (u.a.) __________________________________

Retinal Imagery Studies

RIS

RIS2

Serum Biomarkers

Multiplex
